# *RPS24* alternative splicing is a marker of cancer progression and epithelial-mesenchymal transition

**DOI:** 10.1038/s41598-024-63976-y

**Published:** 2024-06-10

**Authors:** Jiyeon Park, Da Hae Nam, Dokyeong Kim, Yeun-Jun Chung

**Affiliations:** 1 Precision Medicine Research Center, College of Medicine, The Catholic University of Korea, 222 Banpo-daero Seocho-gu, Seoul, 137-701 Republic of Korea; 2 Department of Microbiology, College of Medicine, The Catholic University of Korea, Seoul, Republic of Korea; 3 Department of Biomedicine and Health Sciences, College of Medicine, The Catholic University of Korea, Seoul, Republic of Korea; 4 Integrated Research Center for Genome Polymorphism, The Catholic University of Korea, Seoul, Republic of Korea

**Keywords:** Ribosomal protein genes, Ribosome, *RPS24*, Microexon, Epithelial–mesenchymal transition, Cancer, Computational biology and bioinformatics, Genetics

## Abstract

Although alternative splicing (AS) is a major mechanism that adds diversity to gene expression patterns, its precise role in generating variability in ribosomal proteins, known as ribosomal heterogeneity, remains unclear. The ribosomal protein S24 (*RPS24*) gene, encoding a ribosomal component, undergoes AS; however, in-depth studies have been challenging because of three microexons between exons 4 and 6. We conducted a detailed analysis of *RPS24* AS isoforms using a direct approach to investigate the splicing junctions related to these microexons, focusing on four AS isoforms. Each of these isoforms showed tissue specificity and relative differences in expression among cancer types. Significant differences in the proportions of these *RPS24* AS isoforms between cancerous and normal tissues across diverse cancer types were also observed. Our study highlighted a significant correlation between the expression levels of a specific *RPS24* AS isoform and the epithelial–mesenchymal transition process in lung and breast cancers. Our research contributes to a better understanding of the intricate regulatory mechanisms governing AS of ribosomal protein genes and highlights the biological implications of *RPS24* AS isoforms in tissue development and tumorigenesis.

## Introduction

Ribosomes, which consist of ribosomal proteins and ribosomal RNAs, are the cellular machinery responsible for translation. Despite their vital role in protein synthesis, recent discoveries have revealed their heterogeneity across tissues and disease conditions^1,2^. With the advances in genome technology enabling an in-depth understanding of transcriptional and translational processes, specialized roles of heterogeneous ribosomes in cancer progression and therapeutic resistance have been uncovered^3,4^. Systematic investigations have been conducted to study the functional impact of each ribosomal component using transcriptional and translational profiling^5,6^. Recent studies using large-scale proteomics data have revealed a poor correlation between the mRNA and protein expression levels of genes encoding ribosomal proteins, implicating the significance of post-transcriptional regulation in ribosome biogenesis^7,8^.

Alternative splicing (AS) is a post-transcriptional regulatory process that allows a single gene to encode multiple transcripts. AS is an important source of gene expression heterogeneity that leads to phenotypic diversity^9^. Genome-wide AS studies have been promoted with large-scale sequencing projects such as The Cancer Genome Atlas (TCGA)^10,11^. Recently, the role of AS in ribosomal heterogeneity has gained attention. A single-cell study identified cell type-specific regulation of AS events in ubiquitously expressed genes, including the ribosomal protein S24 (*RPS24*)^12^. A recent study showed that AS can modulate ribosomal composition in glioblastoma cells, suggesting the prognostic and therapeutic value of AS in the ribosomal protein L22-like1 (*RPL22L1*) gene^13^. These studies suggest that AS is an important source of ribosomal heterogeneity.

We recently developed a pan-cancer database of AS events for the molecular classification of cancers (AS-CMC)^14^. Within this repository, *RPS24* AS is one of the most notable AS events, displaying subtype-specific differences across multiple cancer types. However, the details of the *RPS24*S AS event remain elusive due to the involvement of three microexons, which have been overlooked in AS research because of their small size (3–30 nucleotides)^15^. In this study, we analyzed splice junctions linked to these microexons to overcome the existing challenges. Using this approach, we defined the detailed profiles of *RPS24* AS isoforms, pinpointing distinct and robust regulatory patterns in cancers. We observed that one specific isoform, enriched in the muscle, is also significantly expressed in normal brain tissue but is lost in glioma, a brain cancer. Furthermore, we uncovered the relevance of an isoform in epithelial–mesenchymal transition (EMT) and validated it using a range of experimental data. Our findings suggest that *RPS24* AS could be a pivotal molecular marker for tracking cancer progression.

## Results

### Definition and classification of *RPS24* AS isoforms

**Figure 1:**
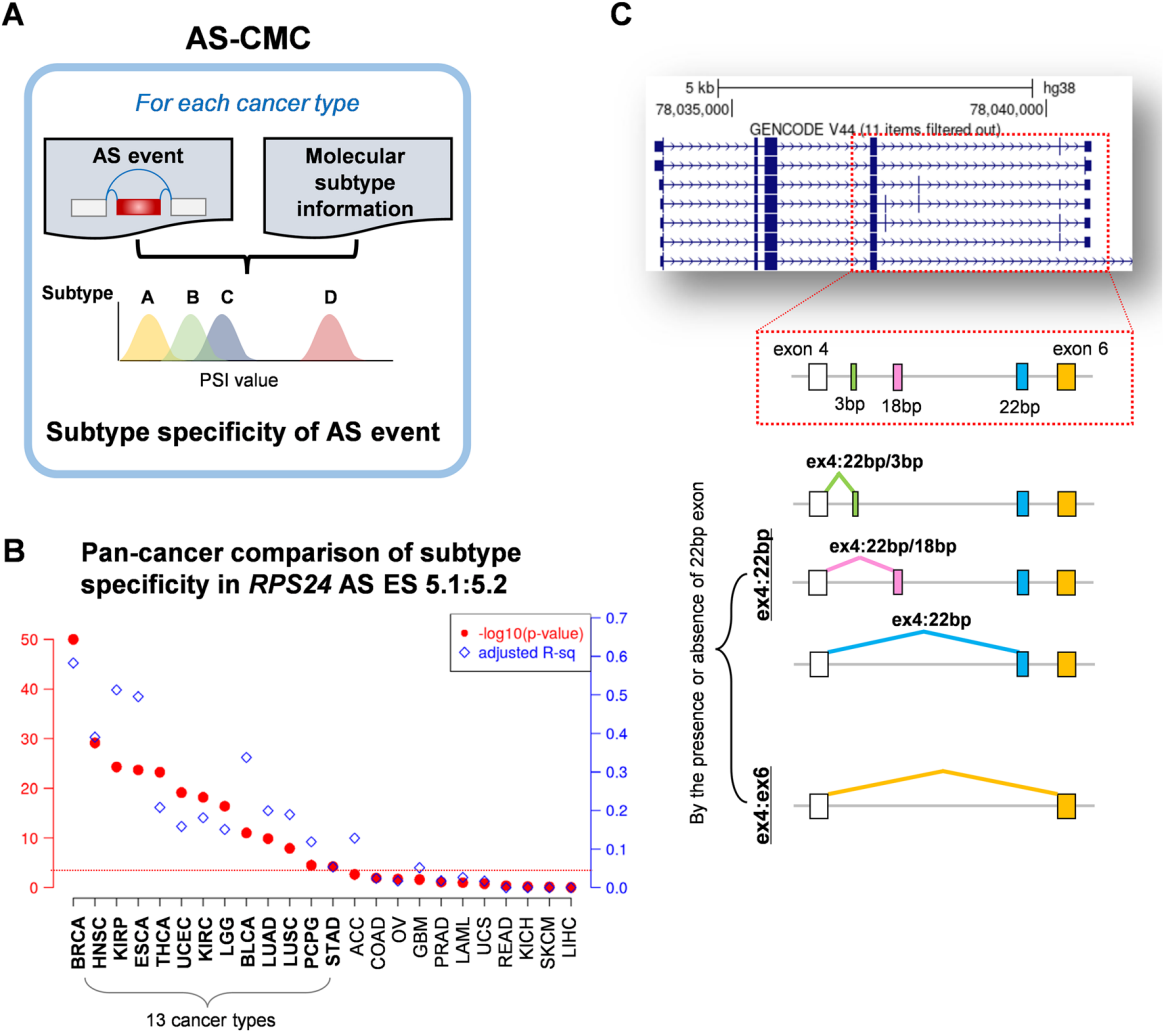
AS analysis of *RPS24* microexons. (**A**) General concept of how an AS event was conducted to test its differences among molecular subtypes in each cancer type in the AS-CMC database. (**B**) Plot demonstrating that an AS event in *RPS24* exhibits discriminative power among molecular subtypes in diverse cancers. Left Y-axis (red scale) represents ANOVA p-value on -log10 scale; right Y-axis (blue scale) represents adjusted R^2^ value. Subtype-specificity was determined to be statistically significant, based on ANOVA p-value threshold of 0.001 (horizontal red line). Significant cancers are marked in bold. ACC: adrenocortical carcinoma. BLCA: bladder urothelial carcinoma. BRCA: breast invasive carcinoma. COAD: colon adenocarcinoma. ESCA: esophageal carcinoma. GBM: glioblastoma multiforme. HNSC: head and neck squamous cell carcinoma. KICH: kidney chromophobe. KIRC: kidney renal clear cell carcinoma. KIRP: kidney renal papillary cell carcinoma. LAML: acute myeloid leukemia. LGG: brain lower grade glioma. LIHC: liver hepatocellular carcinoma. LUAD: lung adenocarcinoma. LUSC: lung squamous cell carcinoma. OV: ovarian serous cystadenocarcinoma. PCPG: pheochromocytoma and paraganglioma. PRAD: prostate adenocarcinoma. READ: rectum adenocarcinoma. SKCM: skin cutaneous melanoma. STAD: stomach adenocarcinoma. THCA: thyroid carcinoma. UCEC: uterine corpus endometrial carcinoma. UCS: uterine carcinosarcoma. (**C**) Classification of *RPS24* AS isoforms. Left panel displays *RPS24* transcript structure within UCSC genome browser, where three microexons are located between exons 4 and 6 of *RPS24*. AS isoforms are grouped into two primary categories based on presence or absence of 22-bp exon. Isoforms lacking the 22-bp exon are designated as “ex4:ex6,” exhibiting a direct connection between exon 4 and exon 6. Isoforms that include the 22-bp exon are further subdivided into three groups based on the nature of the exon preceding the 22-bp exon. If a 3-bp exon precedes the 22-bp exon, isoform is labeled as “ex4:22 bp/3 bp.” In cases where an 18-bp exon is present, isoform is termed “ex4:22 bp/18 bp.” If exon 4 is directly followed by the 22-bp microexon, isoform is termed “ex4:22 bp.” For each isoform, junction reads derived from exon 4 were used to determine their classification. Exons and junctions are color-coded and have distinct names to visualize diversity of *RPS24* AS isoforms. In the UCSC browser, exons are shown as boxes and introns as lines. Within exons, coding regions are shown as thicker boxes and noncoding regions as thinner boxes.

We previously developed a database, named AS-CMC, for identifying subtype-specific AS events for each cancer type (Fig. 1A). In the AS-CMC, an AS event in *RPS24* was significantly associated with molecular subtypes in 13 cancer types (analysis of variance [ANOVA], *p* < 0.001) (Fig. 1B). The *RPS24* gene features three microexons (3, 18, and 22 bp) situated between the constitutive exons (exons 4 and 6) (Fig. 1C). We found that the *RPS24* AS isoforms could be divided into two main groups based on the presence or absence of a 22-bp microexon. If no microexon was present between exons 4 and 6, the isoform was labeled as “ex4:ex6.” Isoforms containing the 22-bp microexon were further classified into three types based on the nature of the preceding exon: “ex4:22 bp/3 bp” if exon 4 was followed by the 3-bp microexon; “ex4:22 bp/18 bp” if exon 4 was followed by the 18-bp microexon; “ex4:22 bp” if exon 4 was directly followed by the 22-bp microexon. For each sample, we quantified the total count of junction reads originating from exon 4, which served as a measure of the overall expression of the *RPS24* gene in that specific sample. Additionally, we determined the proportion of junction reads corresponding to each of these defined isoforms, providing insights into the gene isoform diversity within the samples.

**Figure 2:**
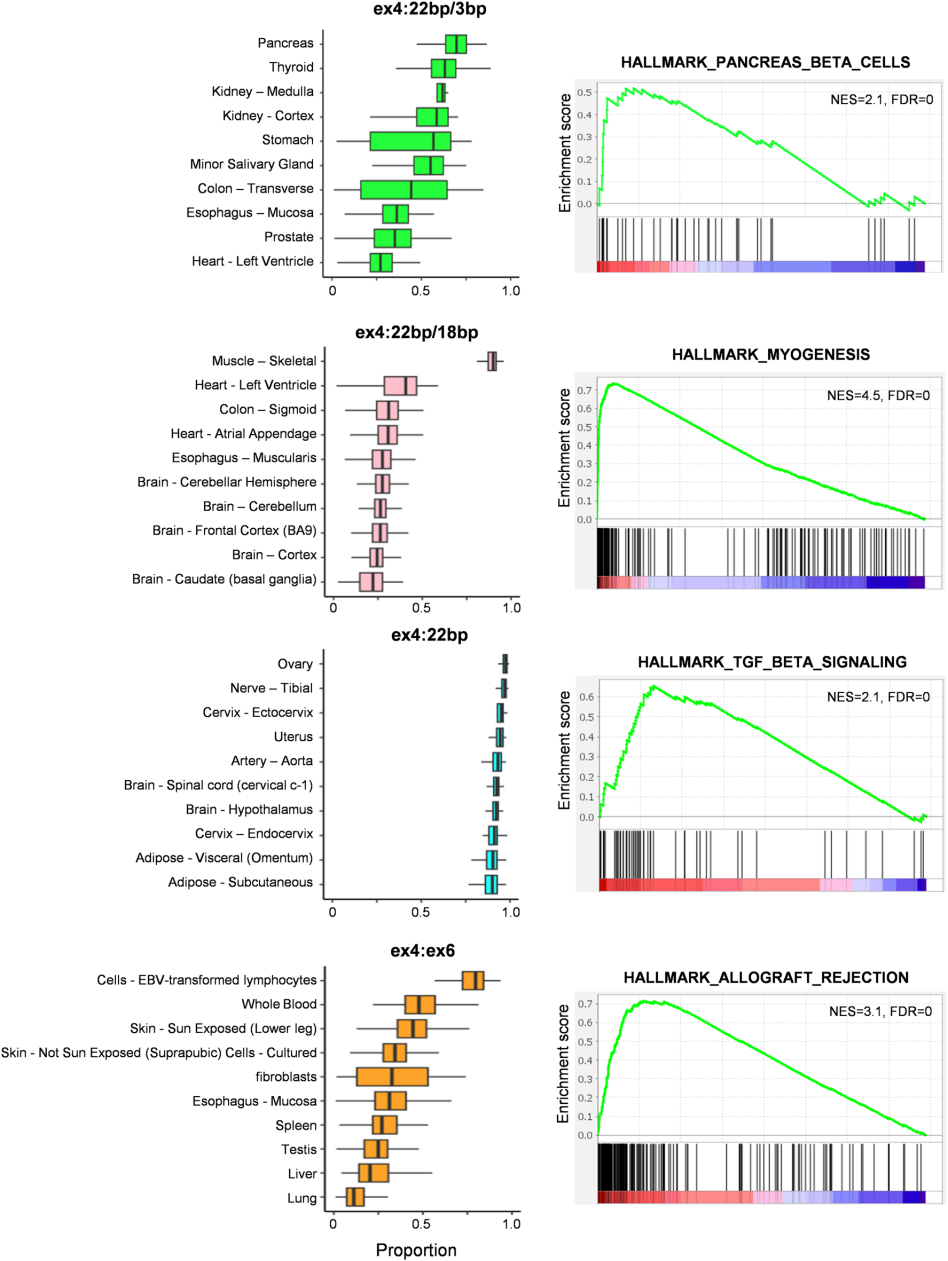
Tissue-specific regulation of *RPS24* AS. (left) Top 10 tissues ranked in order of highest ratio of each splice isoform. Results across all tissues in GTEx data are shown in Supplementary Figs. 2–5. (right) A biological pathway associated with genes highly correlated with usage of each splice isoform. Y-axis represents enrichment score (ES), which indicates the extent to which a gene set is over-represented among top-ranked genes. Normalized enrichment score (NES) and false discovery rate (FDR) values are indicated on the plot. Comprehensive GSEA results are available in Supplementary Tables 5–8, providing additional details on pathways and their significance associated with each isoform.

By applying this approach, we first investigated the prevalence of each *RPS24* AS isoform across various tissues using the Genotype-Tissue Expression (GTEx) data (Supplementary Table 1). Indeed, 99.9% of junction reads involving exon 4 in the GTEx data were categorized into the four types defined above. The ex4:22 bp junction was the most common in all GTEx tissue samples (61%), followed by ex4:22 bp/3 bp (15%), ex4:ex6 (14%), and ex4:22 bp/18 bp (10%) (Supplementary Fig. 1). We then investigated the diversity of *RPS24* AS across tissues, employing the GTEx data. We calculated the tissue-specific enrichment of each isoform and plotted the percentage presence for the top 10 tissues (Supplementary Figs. 2–5) (Fig. 2, left). Ex4:22 bp/3 bp was notably abundant in the pancreas, thyroid gland, and kidneys; ex4:22 bp/18 bp was highly prevalent in the muscle tissue (constituting 90% of all transcripts); ex4:ex6 was prominently expressed in lymphoblastoid cell lines, and ex4:22 bp was abundant in various tissues, including female reproductive organs.

Next, we aimed to understand the biological context of each *RPS24* AS isoform by correlating the proportion of each AS isoform with the expression levels of all other genes. To achieve this, we conducted a gene set enrichment analysis (GSEA) using a pre-ranked list of genes ordered by correlation coefficient. Our objective was to evaluate the enrichment of a specific set of genes within those correlated with each AS isoform. The results were aligned with the distinctive characteristics of the top-ranked tissue types for each *RPS24* AS isoform (Supplementary Tables 5–8). For instance, genes whose expression correlated with the ex4:22 bp/18 bp inclusion were associated with myogenesis (normalized enrichment score = 4.5, false discovery rate = 0), consistent with the high prevalence of this junction in the muscle tissue. Genes linked to ex4:22 bp exhibited enrichment in transforming growth factor beta (TGFβ) signaling or EMT (Fig. 2, right).

### Cancer-specificity of *RPS24* AS isoforms

**Figure 3:**
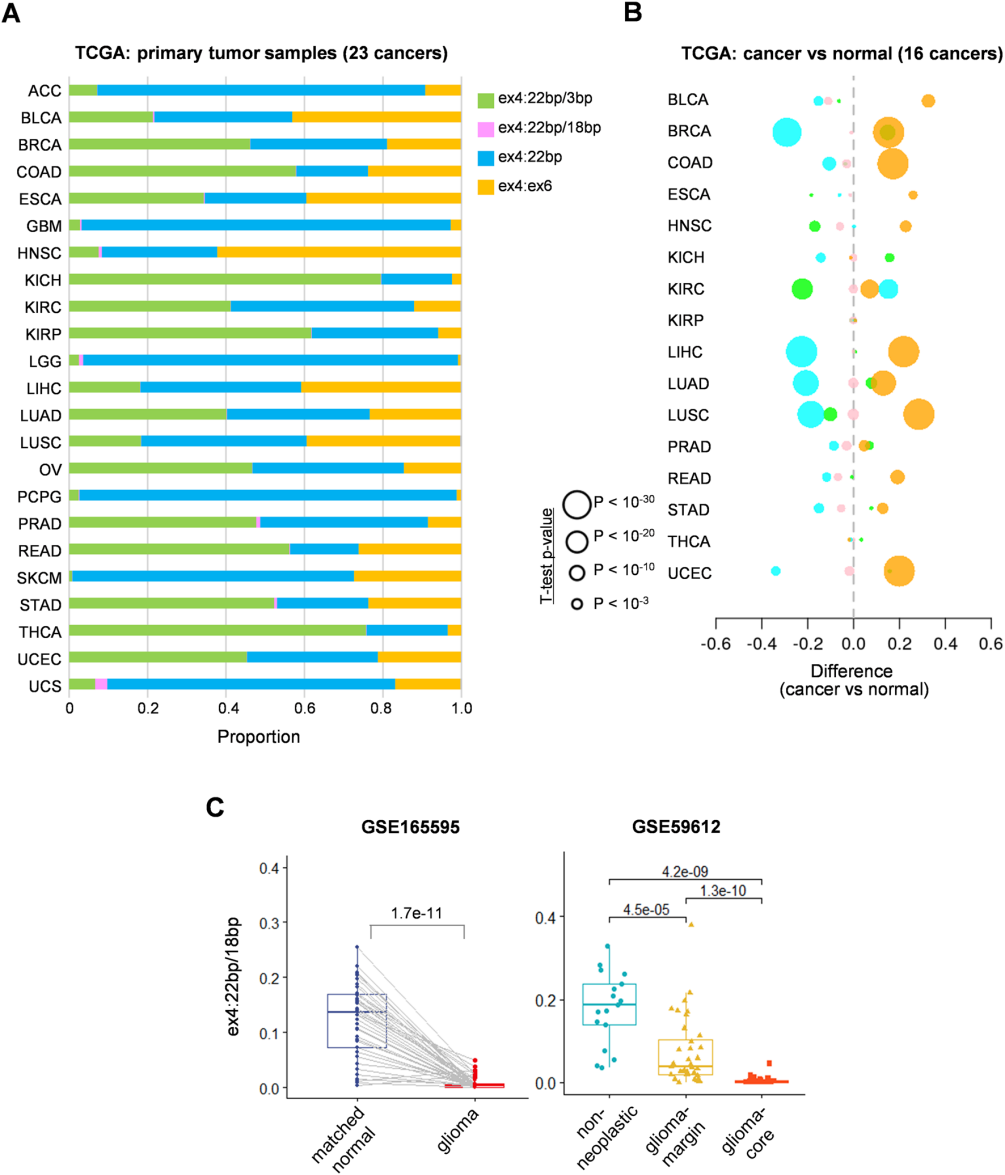
Cancer-specific *RPS24* AS. (**A**) Average proportion of each isoform in 23 cancer types in TCGA. Proportion of each isoform is marked by color (**B**) Cancer-specific regulation of each isoform. X-axis indicates the difference in average proportion of each isoform between cancer and normal samples. Dot size indicates significant difference between cancer and normal tissues. (**C**) Comparison of expression of ex4:22 bp/18 bp isoforms between glioma and normal samples. GSE165595 data contained 41 glioma and matched normal samples^16^. GSE59612 data comprised 92 glioma core and matched margin samples^17^. Surgically removed non-neoplastic brain tissue samples were used as controls. Comparisons between groups were performed using t-test.

Next, we compared the average proportion of each *RPS24* isoform in different cancer types (23 cancer types in TCGA and 10 in CPTAC). The proportion of each isoform differed depending on the cancer type in TCGA data (Fig. 3A). For example, the proportion of the ex4:22 bp isoform was high (average > 0.7) in brain, adrenal, skin, and uterine cancers (LGG, GBM, ACC, SKCM, and UCS), whereas that of the ex4:22 bp/3 bp isoform was high (> 0.6) in kidney and thyroid cancers (KICH, KIRP, and THCA) (Supplementary Fig. 6A). The proportion of ex4:ex6 showed relatively small differences among different cancer types, exhibiting the highest proportion in head and neck squamous cell carcinoma. The ex4:22 bp/18 bp isoform was observed at extremely low levels in all cancer types in TCGA (0–3%). TCGA profiles were largely consistent with those of CPTAC, suggesting the reliability of the characteristics identified in this study, although only 10 cancer types were available in CPTAC (Supplementary Fig. 6B).

To examine cancer-specific changes, we next compared the distribution of *RPS24* AS isoforms between the normal and cancerous tissues in the 16 TCGA cancer types for which normal samples were available (Fig. 3B). Overall, the proportion of ex4:22 bp was lower in cancer tissues than that in normal tissues, whereas the proportion of ex4:ex6 was relatively higher in cancer tissues across most of the 16 cancer types. For instance, in lung cancers (lung adenocarcinoma and lung squamous cell carcinoma), the proportion of ex4:22 bp was significantly lower and that of ex4:ex6 was higher in cancer tissues than that in normal tissues (*p* < 10^–20^, Supplementary Fig. 7).

While the ex4:22 bp/18 bp isoform was prevalent (20%–30%) in GTEx normal brain tissue (Supplementary Fig. 3), it was nearly absent (< 2%) in brain cancers (GBM and LGG) in both TCGA and CPTAC datasets (Supplementary Fig. 6). Because TCGA and CPTAC have very little normal brain tissue for comparison, we verified this trend using other publicly available datasets containing both normal and glioma samples. Analysis of the GSE165595 dataset revealed that ex4:22 bp/18 bp accounted for approximately 15% of normal brain tissue but exhibited a dramatic decline in cancerous tissue (Fig. 3C, left)^16^. Similarly, in the GSE59612 dataset, not only did we observe the same pattern in normal tissues but also within cancerous tissues, where the expression of ex4:22 bp/18 bp decreased more in the tumor center than in the periphery (Fig. 3C, right)^17^. These results suggest that the expression of ex4:22 bp/18 bp may be involved in the development of brain cancer.

### *RPS24* AS is potential marker for EMT

**Figure 4:**
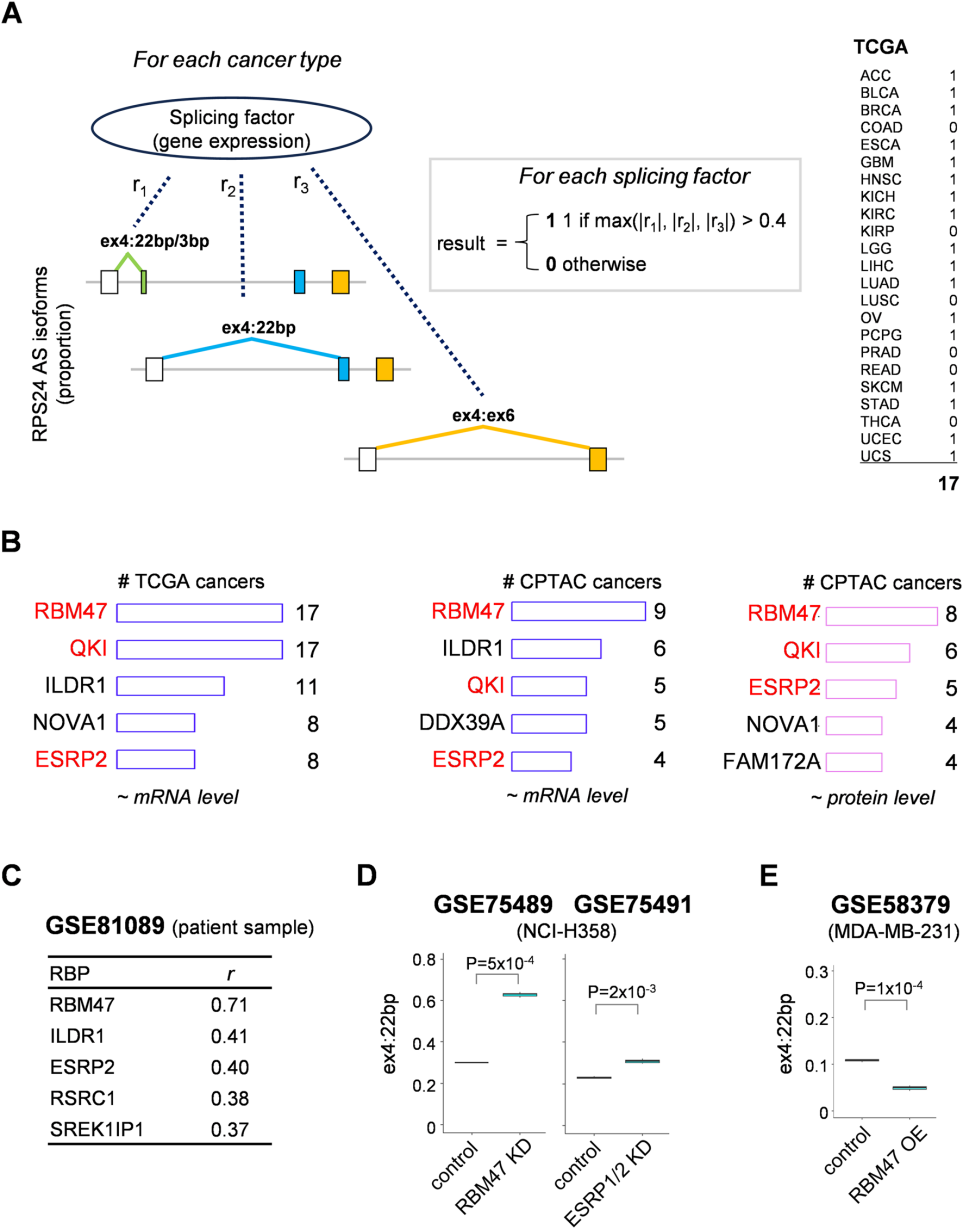
Association of *RPS24* AS with EMT-related splicing factors. (**A**) General concept of identifying splicing factors associated with *RPS24* AS isoforms. For each splicing factor, we analyzed the correlation of gene expression (mRNA level) of splicing factors with *RPS24* isoform ratios. A value of 1 was assigned if maximum absolute correlation coefficient exceeded 0.4, indicating a significant relationship in the given cancer type. This analysis was repeated for all cancer types; those showing a significant association were listed. Regarding splicing factor gene expression, mRNA levels were used for TCGA data, while both mRNA and protein levels were considered for CPTAC data. (**B**) Top five splicing factors demonstrating a significant correlation with *RPS24* AS isoforms in multiple cancer types are shown. Number on right side of each bar indicates number of cancer types where a significant correlation (|r|> 0.4) was observed. Splicing factors that consistently showed top 5 frequency of significant correlation with diverse cancer types across all three datasets are highlighted in red. (**C**) Top five splicing factors demonstrating a significant correlation with *RPS24* AS isoforms in lung cancer dataset (GSE81089)^20^. (**D**) Proportion of ex4:22 bp related to knockdown of *RBM47* (GSE75489) and *ESRP* (GSE75491) in lung cancer cell line NCI-H358^21^. Ex4:22 bp proportion was significantly elevated in *RBM47-*knockdown cells and slightly elevated in *ESRP1/2*-knockdown cells compared to that in control cells. (**E**) Proportion of ex4:22 bp related to overexpression of *RBM47* (GSE58379) in breast cancer cell line MDA-MB-231^22^.

Next, we aimed to identify the splicing factors associated with *RPS24* AS during tumorigenesis. We calculated the correlation between the proportion of *RPS24* AS isoforms and the mRNA expression of RNA-splicing factors in TCGA and CPTAC databases (Fig. 4A). Since splicing genes regulate their own splicing more dynamically, there is often a mismatch between mRNA and protein expression^18,19^. Therefore, we included not only the mRNA levels of these splicing factors but also the protein levels. We defined a threshold for the correlation coefficient and applied it to a maximum of three values. If the correlation met the specified threshold (|r|> 0.4) for a specific cancer type, we assigned a value of 1 to indicate a significant correlation; otherwise, a value of 0 indicated no significant correlation (an example is shown on the right side of Fig. 4A).

We examined the number of cancers in which each splicing factor exhibited a significant correlation with the proportion of *RPS24* AS isoforms (Fig. 4B). Among the splicing factor genes, *RBM47* consistently ranked at the top of the list for both mRNA and protein levels, followed by *QKI* and *ESRP2*, indicating the potential regulatory roles of splicing factors in *RPS24* AS isoform expression in diverse cancer contexts. Another RNA-seq dataset (GSE81089) encompassing 199 patients with lung cancer showed a consistent correlation between *RPS24* AS and *RBM47* expression (Fig. 4C)^20^.

To obtain direct evidence, we examined the response of *RPS24* AS to different expression levels of *RBM47* in cancer cell lines. The proportion of ex4:22 bp was significantly increased in response to *RBM47* knockdown, with a more pronounced effect than that in response to the knockdown of *ESRP1* and *ESRP2* (Fig. 4D)^21^. Conversely, the proportion of ex4:22 bp significantly decreased upon *RBM47* overexpression (Fig. 4E)^22^. These results imply that *RPS24* AS could be a target of the splicing factor *RBM47*, suggesting a potential influence of *RBM47* on *RPS24* AS.

**Figure 5:**
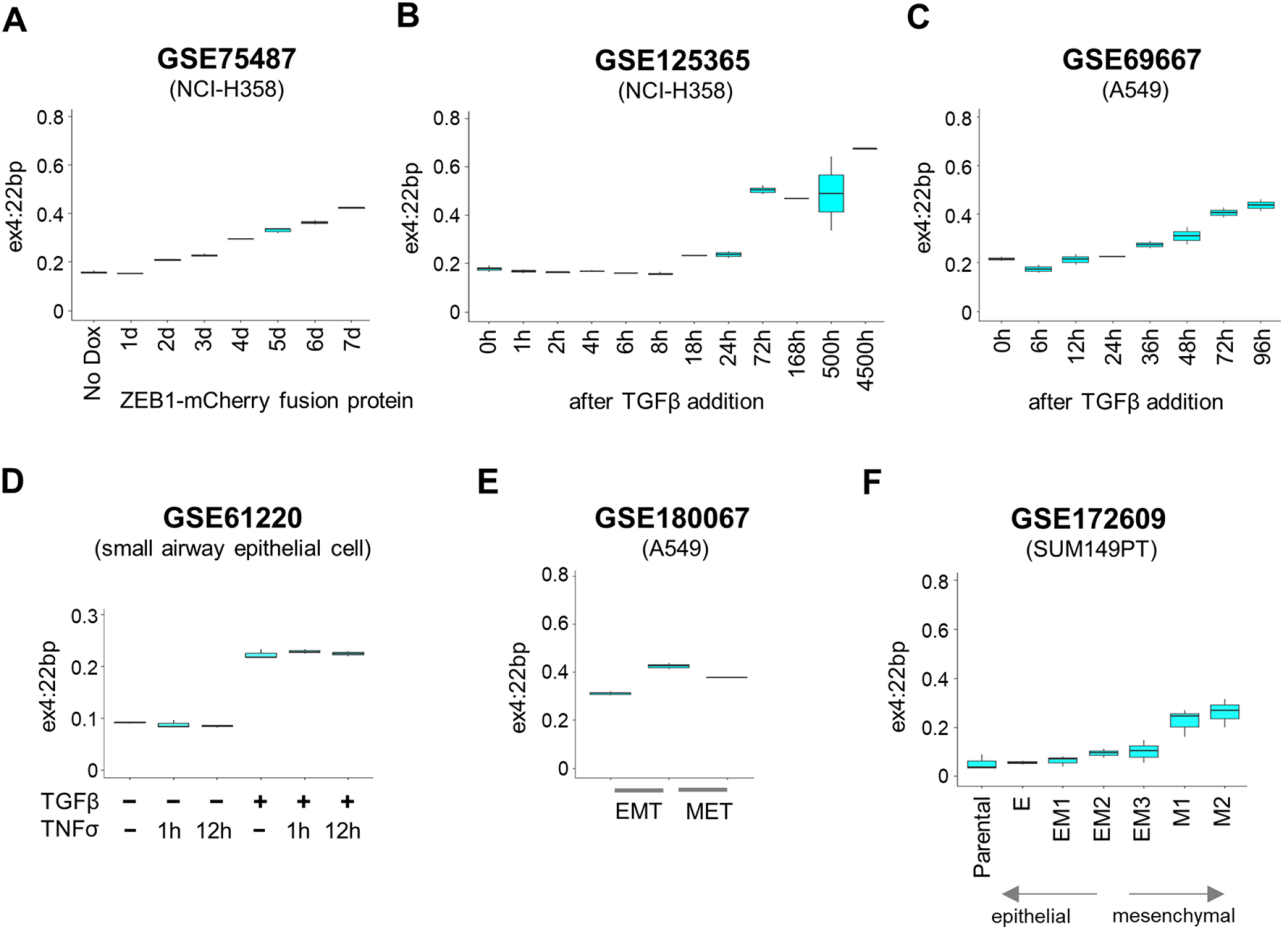
Experimental evidence in association of *RPS24* AS with EMT. (**A**) Changes in ex4:22 bp proportion over time during EMT progression in NCI-H358 cell line by ZEB1 expression (GSE75487)^21^. (**B**, **C**) Changes in ex4:22 bp proportion over time during EMT progression in NCI-H358 and A549 cell lines by TGFβ-induction (GSE125365 and GSE69667)^25,26^. X-axis represents time after ZEB1 or TGFβ treatment. (**D**) Changes in ex4:22 bp proportion under TGFβ or TNFα stimulation in small airway epithelial cells (GSE61220)^27^. (**E**) Changes in ex4:22 bp proportion during EMT-MET plasticity in A549 cells (GSE180067)^28^. EMT was induced via TGFβ and EGF treatment, and reversal to MET was induced by removing those cytokines from the medium. (**F**) Changes in ex4:22 bp proportion during EMT-MET plasticity in breast cancer cell line (SUM149PT) (GSE172609)^29^. There are six single-cell-derived clonal populations ranging from epithelial-like (E) to mesenchymal (M1 and M2), which included three intermediate states (EM1, EM2, and EM3).

Considering the roles of splicing factors, such as *RBM47*, *QKI*, and *ESRP*2, in EMT^21,23,24^, we further investigated the intricate relationship between *RPS24* AS and EMT by analyzing various RNA-seq datasets obtained from experimental EMT models^21,25,26^. Although these EMT models were independently constructed, they consistently demonstrated a progressive increase in ex4:22 bp expression during EMT induction (Fig. 5A–C)^21,25,26^. This trend was observed regardless of whether the models were driven by zinc finger E-box binding homeobox 1 (ZEB1) or TGFβ. While the increase in ex4:22 bp expression was notable upon stimulation with TGFβ, it remained unresponsive to treatment with tumor necrosis factor alpha (TNFα) in airway epithelial cells (Fig. 5D)^27^.

TNFα is another cytokine associated with inflammation and immune responses, similar to TGFβ. This contrast suggests that TNFα and TGFβ exhibit distinct mechanisms of action and effects on *RPS24* AS. Ex4:22 bp exhibited an incremental response to the stimulation of TGFβ and epidermal growth factor (EGF), actively promoting the EMT process. Conversely, it exhibited a reverse pattern upon withdrawal of these cytokines, facilitating the mesenchymal-epithelial transition (MET) (Fig. 5E)^28^. The consistent response of ex4:22 bp during EMT was corroborated in a metastatic breast cancer cell line (SUM149PT), where its behavior closely mirrored the EMT status (Fig. 5F)^29^. These findings substantiate the potential of *RPS24* AS as a molecular marker of cancer cell plasticity.

### Experimental validation of *RPS24* AS changes in EMT

**Figure 6:**
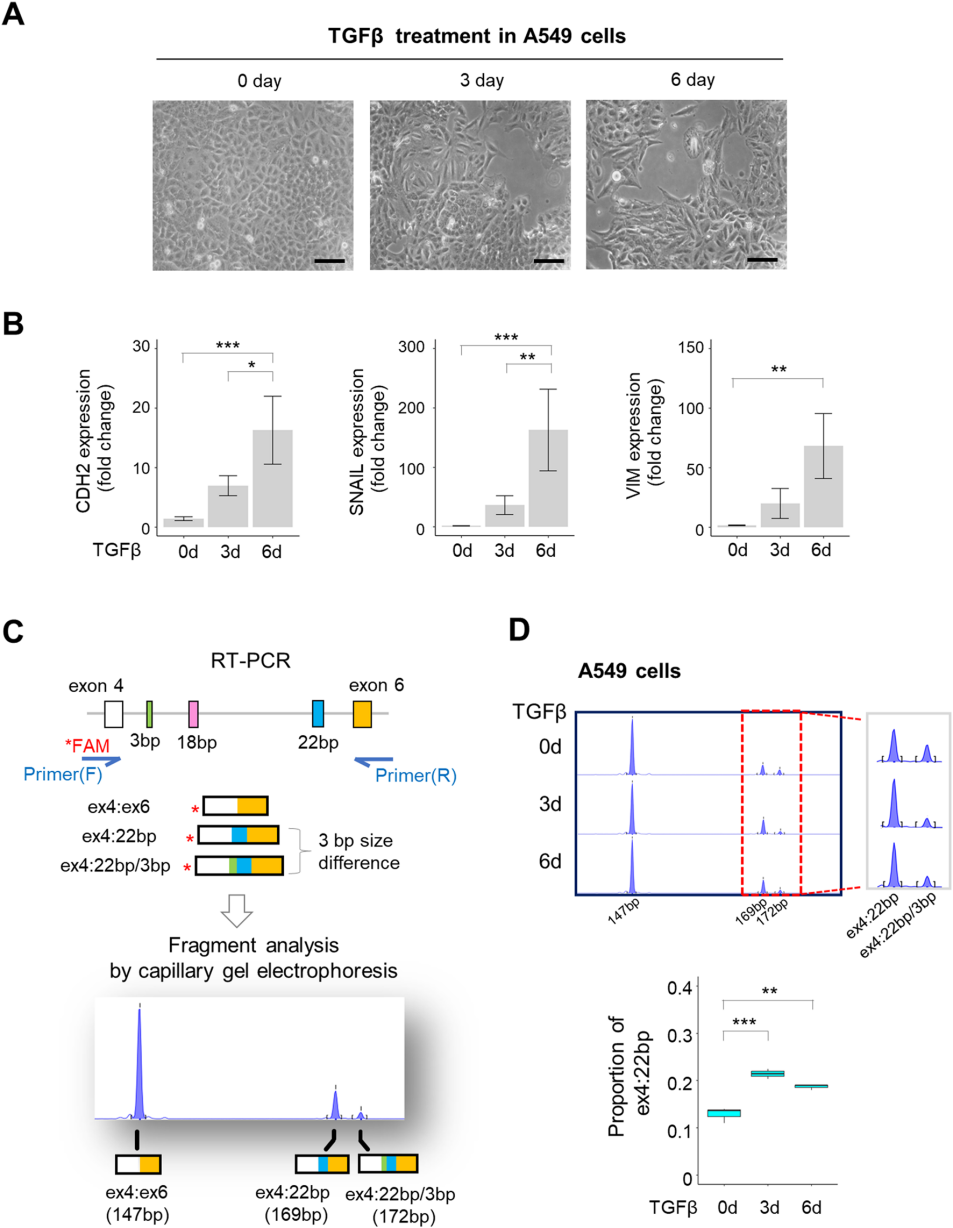
Experimental validation of ex4:22 bp increase upon EMT induction in A549 cells. (**A**) Bright-field images depicting the changes in the cellular morphology of A549 cells after 3 and 6 days of treatment with TGFβ1 (10 ng/mL) (magnification: 200X, scale bar: 100 µm). (**B**) Changes in the expression of EMT marker genes. qPCR was conducted on untreated control and TGFβ1-treated A549 cell lines. GAPDH was used as the endogenous control, and the relative gene expression was presented as the 2^−ΔΔCT value^. The results are presented as mean ± SEM and were analyzed using ANOVA/Tukey HSD (*p < 0.05, **p < 0.01, ***p < 0.001). (**C**) Fragment analysis of fluorescently labeled PCR products. The primers were designed to amplify the region between exons 4 and 6 (indicated by blue arrows). The forward primer was labeled with 6-FAM dye, and the PCR products were separated using capillary electrophoresis at a 1:100 dilution. (**D**) Fragment analysis of *RPS24* AS isoforms in A549 cells undergoing EMT. Electropherograms representing *RPS24* AS isoforms are displayed under three conditions. Capillary electrophoresis was repeated three times for each condition, and one representative result is shown. The peaks corresponding to ex4:22 bp and ex4:22 bp/3 bp isoforms were magnified and shown on the right. The area of each peak was quantified as the expression of each isoform, and each isoform's proportion of the total expression was calculated. The boxplot below illustrates the changes in the proportion of ex4:22 bp during EMT induction. *p* values were derived from ANOVA/Tukey HSD (*p < 0.05, **p < 0.01, ***p < 0.001).

In addition to analyzing published data, we further confirmed the changes in *RPS24* AS experimentally by inducing EMT in the lung cancer cell line A549. After 3 and 6 days of TGFβ1 treatment, we observed that the cells exhibited a spindle-shaped mesenchyme-like morphology (Fig. 6A). Using quantitative polymerase chain reaction (qPCR), we confirmed the increased RNA expression of EMT marker genes (Fig. 6B). To calculate the ex4:22 bp ratio among all *RPS24* AS isoforms, it was important to separate it from the ex4:22 bp/3 bp isoform, which differs in length by only 3 bp. Thus, we performed capillary electrophoresis to separate the isoforms based on their length difference as described by Olivieri et al.^12^. We amplified the region containing *RPS24* AS using primers labeled with fluorescein (FAM) and subjected this fluorescently labeled product to capillary electrophoresis. We then analyzed its peaks to calculate the ratio of each of the three isoforms (Fig. 6C). Upon comparison of the electropherograms, we observed a significant increase in the ex4:22 bp peak upon EMT induction, which was also significant when we calculated the ratio of the isoforms (*p* < 0.01, Fig. 6D).

## Discussion

*RPS24* encodes a component of the small 40S ribosomal subunit. With the advent of high-throughput sequencing technology, it has been apparent that *RPS24* contains three consecutive microexons at its 3′ end. These exons combine in various ways to produce diverse RNA isoforms^12^. However, the functional consequences of these changes, especially their implications for tumorigenesis, are not yet understood. In our pan-cancer AS database^14^, *RPS24* AS was significantly associated with molecular subtypes of diverse cancers, suggesting its potential involvement in tumorigenesis.

Previous reports regarding *RPS24* AS have focused primarily on the differential use of a 22-bp exon; AS events with the other two alternative exons (3- and 18-bp exons) are less well-represented because of their shorter length^30,31^. Through a detailed analysis of the junction reads originating from exon 4, we identified four *RPS24* AS isoforms from normal human tissue transcriptome data^32^ and their profiles: ex4:22 bp (61%); ex4:22 bp/3 bp (15%); ex4, ex6 (14%); and ex4:22 bp/18 bp (10%).

Recently, Olivieri et al. demonstrated AS events that can define tissue compartments and cell types using 10X single-cell analysis^12^. While Olivieri et al. reported the cell type-specific expression of *RPS24* AS isoforms in 12 human tissues, our study significantly extended this work by providing a comprehensive overview of isoform ratios across more than 50 human tissues. In the case of the ex4:22 bp/3 bp isoform, Olivieri et al.’s work emphasized its predominant expression in human epithelial cells. However, we report this isoform in substantial amounts in the pancreas, thyroid, and kidney tissues (Fig. 2). These tissues were not included in Olivieri et al.'s study. This finding suggests that the ex4:22 bp/3 bp isoform would be highly expressed not only in epithelial cells but also among hormone-producing and secretory cells.

As *RPS24* AS varies depending on cell type^12^, the changes in *RPS24* AS isoform distribution observed in different tissue types may reflect differences in cellular composition between tissues. Therefore, we focused more on comparing normal and cancerous tissues within one organ. During this process, we carefully compared GTEx normal tissue data with TCGA/CPTAC cancer tissue data, which led to the discovery of differences in the ex4:22 bp/18 bp isoform in brain cancer. Ex4:22 bp/18 bp has been reported to be muscle-specific^12^, and our observation aligns with this finding (Fig. 2). Additionally, our analysis revealed the presence of the ex4:22 bp/18 bp isoform in normal brain tissues, constituting 20–30% of the total *RPS24* expression (Fig. 2). However, the isoform was expressed at an extremely low level in brain tumor tissues and showed substantially reduced expression in the peripheral area of the tumor (Fig. 3). Based on findings reported by Uniacke’s lab regarding the increased expression of ex4:22 bp isoforms under hypoxic conditions^31,33^, we hypothesize that the observed difference in the expression of ex4:22 bp/18 bp between the center and periphery of brain tumors may also be attributed to hypoxic regulation. Further large-scale analyses and functional studies are required to verify this hypothesis.

The most interesting finding of our study was the association between the ex4:22 bp isoform and EMT, a crucial cellular process implicated in cancer metastasis. This connection was inferred through a comprehensive analysis of splicing factors correlated with *RPS24* AS. We substantiated the validity of this finding through a series of experiments conducted on various cell lines. In our analysis, the ex4:22 bp isoform ratio increased as EMT progressed and decreased during the reverse process MET. This observation suggests that *RPS24* AS is closely associated with cellular plasticity, which plays a crucial role in dynamic cellular transformations. Recent reports have emphasized the involvement of ribosomal biogenesis in EMT^3,34^. EMT-related ribosome production coincides with G1/S cell cycle arrest^35^. Recently, targeting ribosome biogenesis has emerged as a novel therapeutic approach to overcome chemoresistance associated with EMT in breast cancer^36^. Considering these findings, further research is needed to elucidate how *RPS24* AS influences ribosome function and structure during EMT.

A limitation of our study is that, although we successfully examined the compositional changes in *RPS24* AS isoforms on a large scale, we could not fully characterize the entire structure of each *RPS24* AS isoform. To explore the complex AS events in the *RPS24* gene in more depth, we should consider using long-read sequencing technologies^37^. Another limitation was that we did not investigate the functional consequences of *RPS24* AS changes. A recent study suggested that proteins derived from the ex4:22 bp isoform exhibit increased stability, potentially promoting hypoxic cell survival and growth^33^. Another study conducted a spatial transcriptomic analysis of mouse organs and revealed the topological regulation of the *Rps24* AS isoforms in brain and kidney tissues, suggesting a crucial role for this isoform in cell fate determination^38^. This evidence suggested that *RPS24* AS isoforms may contribute to the creation of heterogeneity in ribosomes, which could in turn alter the ribosome’s translational properties and consequently affect tumorigenesis. Further in-depth functional studies on various *RPS24* AS isoforms will be required to elucidate their distinctive features.

In conclusion, in this study, we uncovered important insights into the tissue-specific and cancer-specific regulation of *RPS24* AS isoforms. To our knowledge, it is the first study to propose an association between the ex4:22 bp isoform and EMT in cancer. Our findings will be useful in understanding the biological implications of *RPS24* AS isoforms in tissue development and tumorigenesis.

## Methods

### Data source

For tissue-specific regulation, we used GTEx data from disease-free tissue sites in approximately 1000 individuals^32^. We downloaded exon-exon junction read counts from the GTEx data portal (https://gtexportal.org/home/datasets). For cancer-specific regulation, we used TCGA and Clinical Proteomic Tumor Analysis Consortium (CPTAC) data. We obtained splice junction quantification data from TCGA and CPTAC from the NCI GDC data portal (https://portal.gdc.cancer.gov/)^39^. Data were accessed using the National Institutes of Health Database of Genotypes and Phenotypes.

For RNA sequencing (RNA-seq) datasets published in the public domain, we downloaded sequencing files from the Sequence Read Archive (SRA) via a search on the Gene Expression Omnibus website (https://www.ncbi.nlm.nih.gov/geo/). The raw data were in SRA file format; therefore, we converted them to fastq files using the SRA toolkit. The fastq files were mapped to the reference genome (hg38) using the STAR aligner in the two-pass mode^40^. We used only the SJ.out.tab files summarizing the splice junctions, one of the STAR output files. The sample information for each dataset is presented in Supplementary Tables 1–4.

### *RPS24* AS analysis using splice junction reads

For each dataset, we combined the splice junction reads on a sample-by-sample basis and extracted reads spanning exon 4 of *RPS24*. In *RPS24*, exon 4 is consistently expressed across all isoforms, and variations in isoforms arise based on subsequent exons. Based on transcriptome databases, we categorized these isoforms into four distinct groups, as shown in Fig. 1. For each sample within the dataset, we quantified the total number of junction reads originating from exon 4. This count served as a measure of the overall expression level of the *RPS24* gene specifically within that particular sample. To assess the isoform proportions, we calculated the ratio by dividing the number of junction reads representing each isoform by the total number of junction reads stemming from exon 4.

### Analysis of gene sets related to *RPS24* AS

To elucidate the biological context associated with each *RPS24* AS isoform, we calculated the correlation between the ratios of each *RPS24* AS isoform and the expression of individual genes. The resulting list of genes, sorted by the correlation coefficients, underwent a pre-ranked GSEA using the hallmark gene sets (H) available in the Molecular Signatures Database (MSigDB)^41,42^. This GSEA pre-ranked test assessed the enrichment of genes from specific gene sets within the top of this ranked list. The enrichment score, a crucial metric, quantifies the over-representation of a gene set among genes positively correlated with the *RPS24* AS isoform usage. The normalized enrichment score provides a robust measure of enrichment while accounting for variations in gene set size and data noise. For each gene set under consideration, we recorded several key metrics, including the number of genes, enrichment score, normalized enrichment score, nominal p-value, and false discovery rate. The results are detailed in Supplementary Tables 5–8.

### Correlation analysis of *RPS24* AS with RNA-splicing factor genes

We compiled a list of genes involved in the human RNA splicing process from MSigDB (GOBP_RNA_SPLICING), encompassing 463 genes. Gene expression data from TCGA dataset were retrieved using TCGAbiolinks R package^43^, whereas gene expression data from the CPTAC dataset were obtained using the CPTAC Python package, accessible at https://pypi.org/project/cptac. To assess the correlation between *RPS24* AS isoforms and the expression levels of splicing factor genes in each cancer type, Pearson’s correlation coefficients were computed. To identify the most relevant splicing factors, we adopted the following criterion: splicing factors were considered significant if their correlation coefficient (|r|) exceeded 0.4. The top five splicing factors were selected based on the number of cancer types that exhibited significant correlations. This approach allowed us to identify the key splicing factors that displayed substantial and consistent associations with *RPS24* AS isoforms across multiple cancer types.

### Experimental validation of changes in *RPS24* AS isoform composition in EMT models

The lung adenocarcinoma cell line A549 (American Type Culture Collection, USA) was cultured in RPMI-1640 medium (HyClone, USA) supplemented with 10% fetal bovine serum (HyClone) and 1% penicillin–streptomycin (Sigma-Aldrich, Germany). The induction of the EMT-like phenotype was achieved by treating cells with 10 ng/mL of TGFβ1 (R&D Systems, USA). The primers used for amplification of EMT marker genes and *RPS24* AS isoforms were previously reported^30,44,45^. EMT marker expression was measured through qPCR, which was conducted using a ViiA™ 7 real-time PCR system (Thermo Fisher Scientific, USA). Each 10 µL PCR reaction contained the reverse transcription product, ROX reference dye, primers, and SYBR Green I dye, employing the THUNDERBIRD™ SYBR™ qPCR mix kit (TOYOBO, Japan). For *RPS24* AS isoforms, forward primers were synthesized using 6-FAM at the 5' end, and PCR was performed using KOD OneTM PCR Master Mix (TOYOBO). The *RPS24* AS PCR product was diluted 1:100 and separated through capillary electrophoresis using an ABI 3130xl Genetic Analyzer (Thermo Fisher Scientific). The size and position of each peak were determined using Peak Scanner software (Thermo Fisher Scientific). The area of each peak was then quantified to determine the expression of each isoform and calculate its proportion of the total expression.

### Supplementary Information

Supplementary Figure 1.

Supplementary Figure 2.

Supplementary Figure 3.

Supplementary Figure 4.

Supplementary Figure 5.

Supplementary Figure 6.

Supplementary Figure 7.

Supplementary Legends.

Supplementary Tables.

## Data Availability

The GTEx data analyzed in this study were obtained from the GTEx portal, TCGA and CPTAC datasets from the GDC data portal, and other validation datasets from RNA-seq data in the GEO database (Supplementary Tables 1–4). Further inquiries can be directed to the corresponding author.
