# Supplementary figures and images for: RPS24 alternative splicing is a marker of cancer progression and epithelial-mesenchymal transition
Source: Sci Rep. 2024 Jun 10;14:13246. doi: 10.1038/s41598-024-63976-y (PMC11162997; doi:10.1038/s41598-024-63976-y)

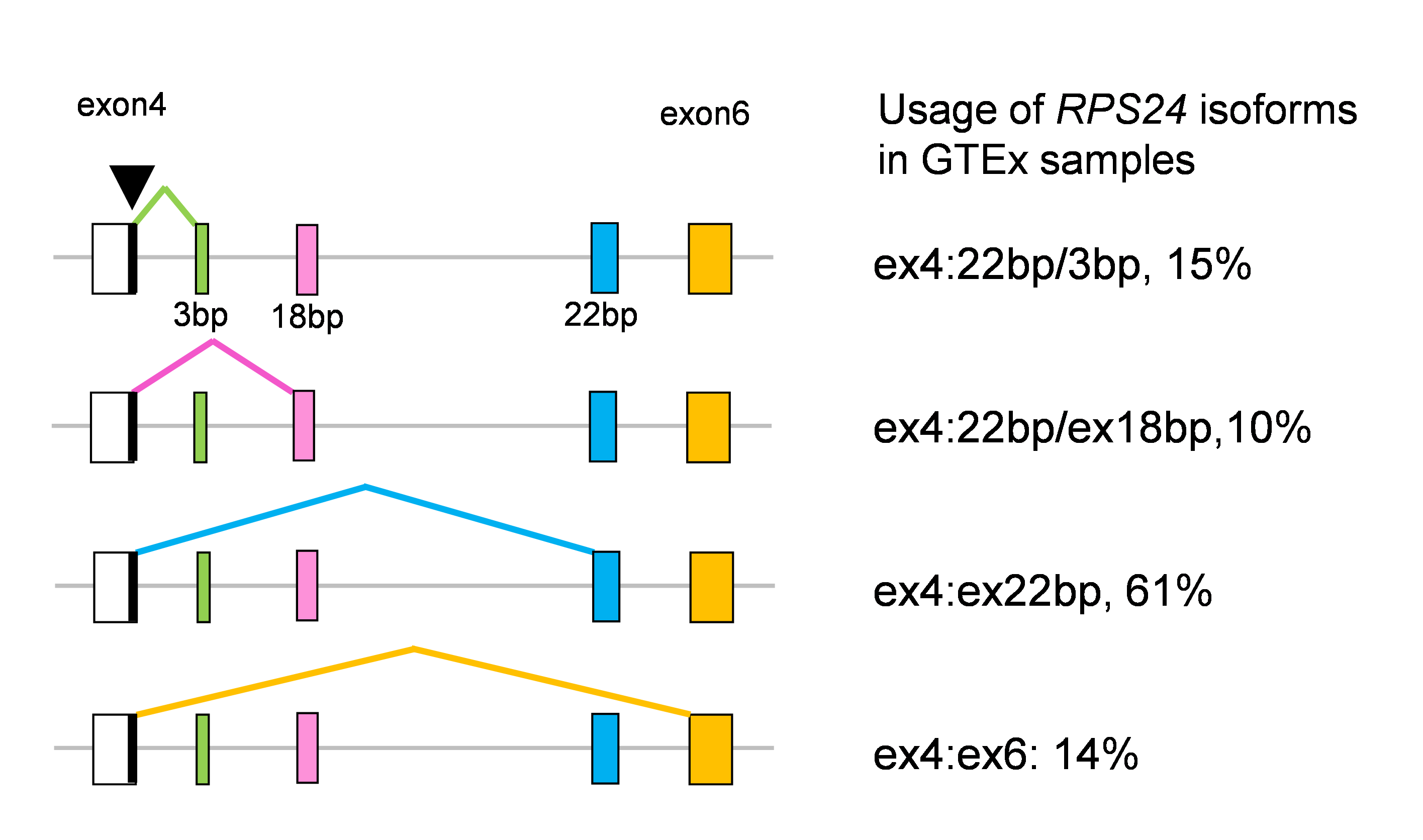

Supplement: Supplementary file 1 — Supplementary Figure 1. [file 41598_2024_63976_MOESM1_ESM.tif]

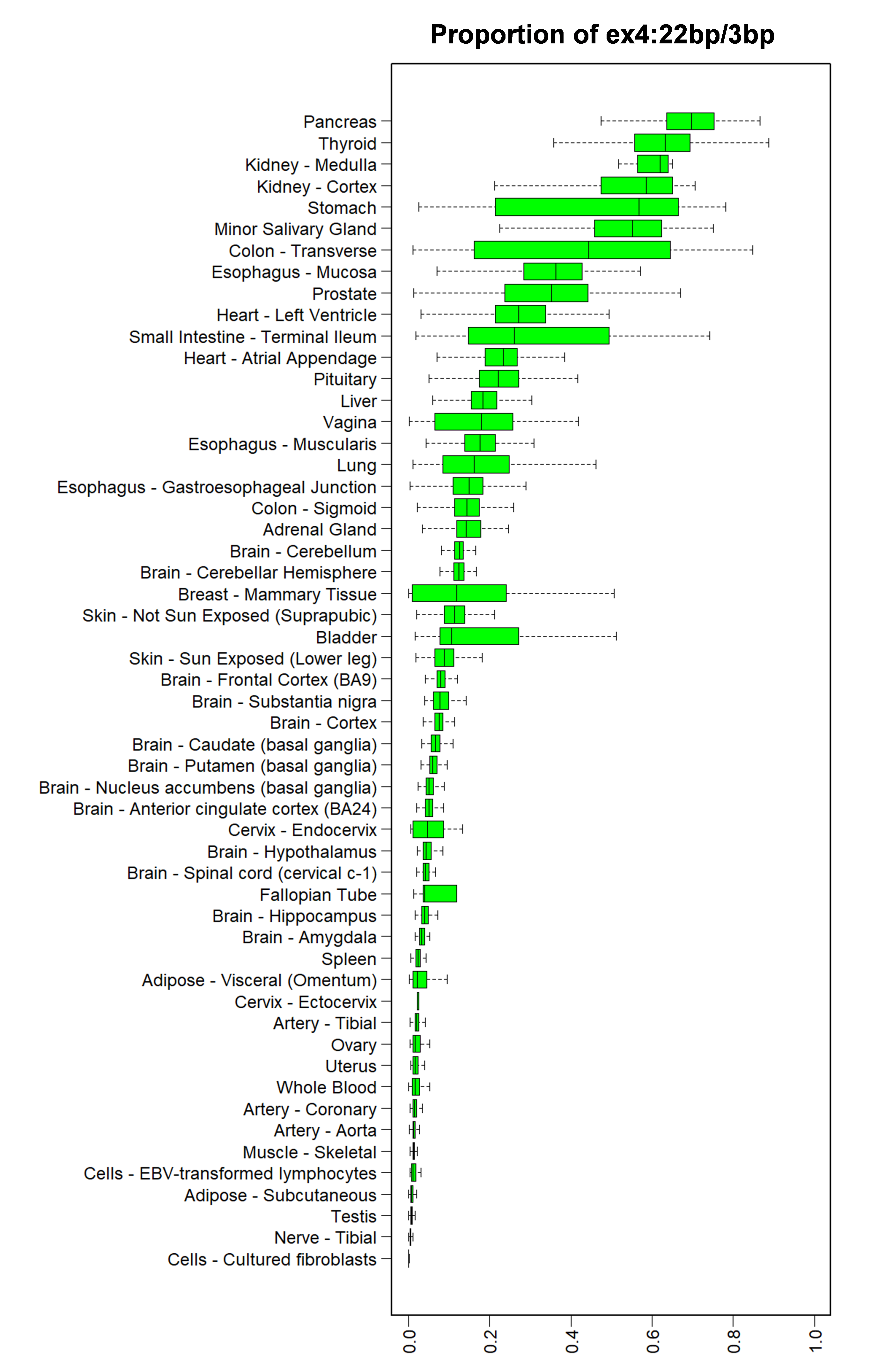

Supplement: Supplementary file 2 — Supplementary Figure 2. [file 41598_2024_63976_MOESM2_ESM.tif]

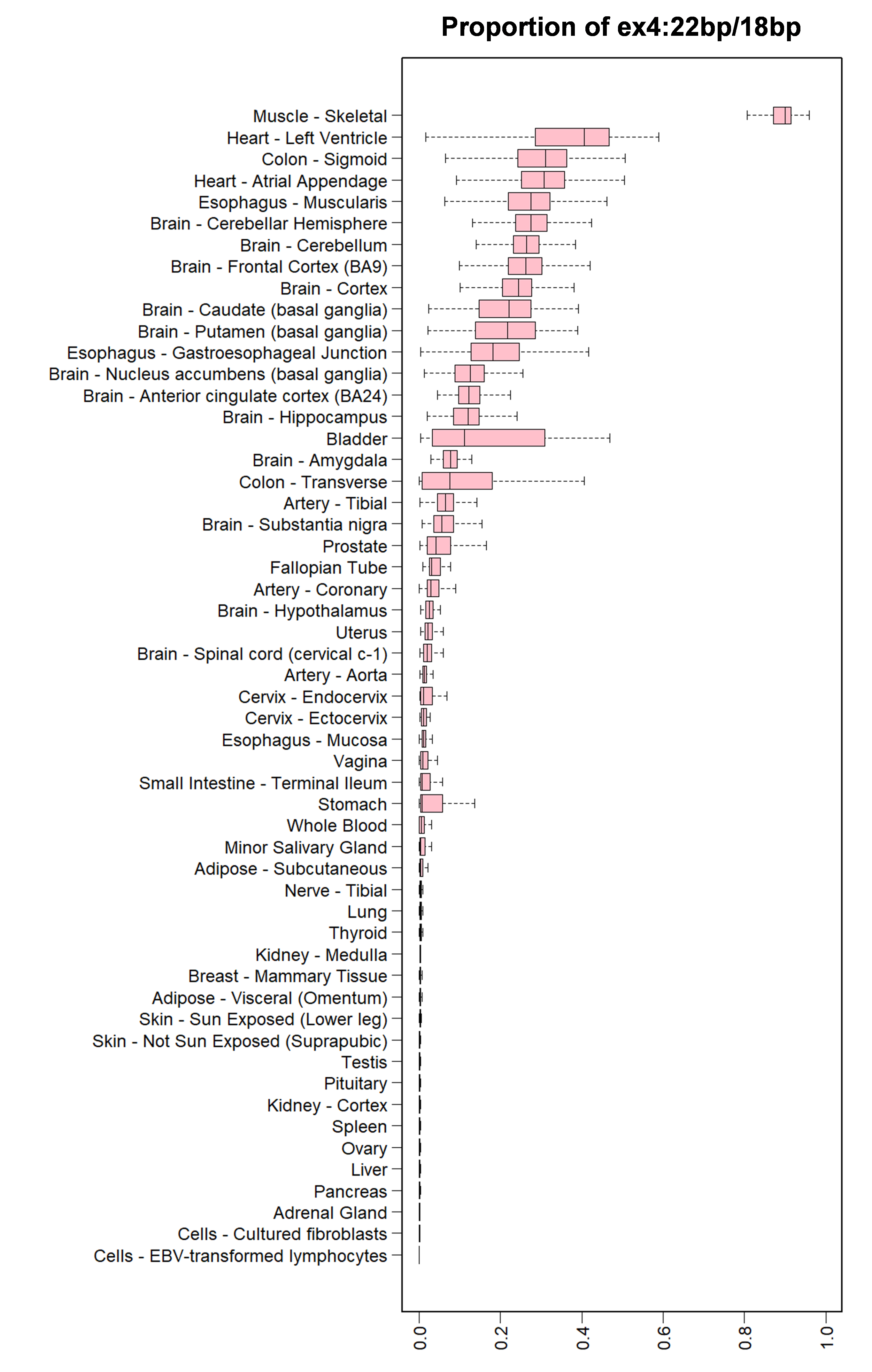

Supplement: Supplementary file 3 — Supplementary Figure 3. [file 41598_2024_63976_MOESM3_ESM.tif]

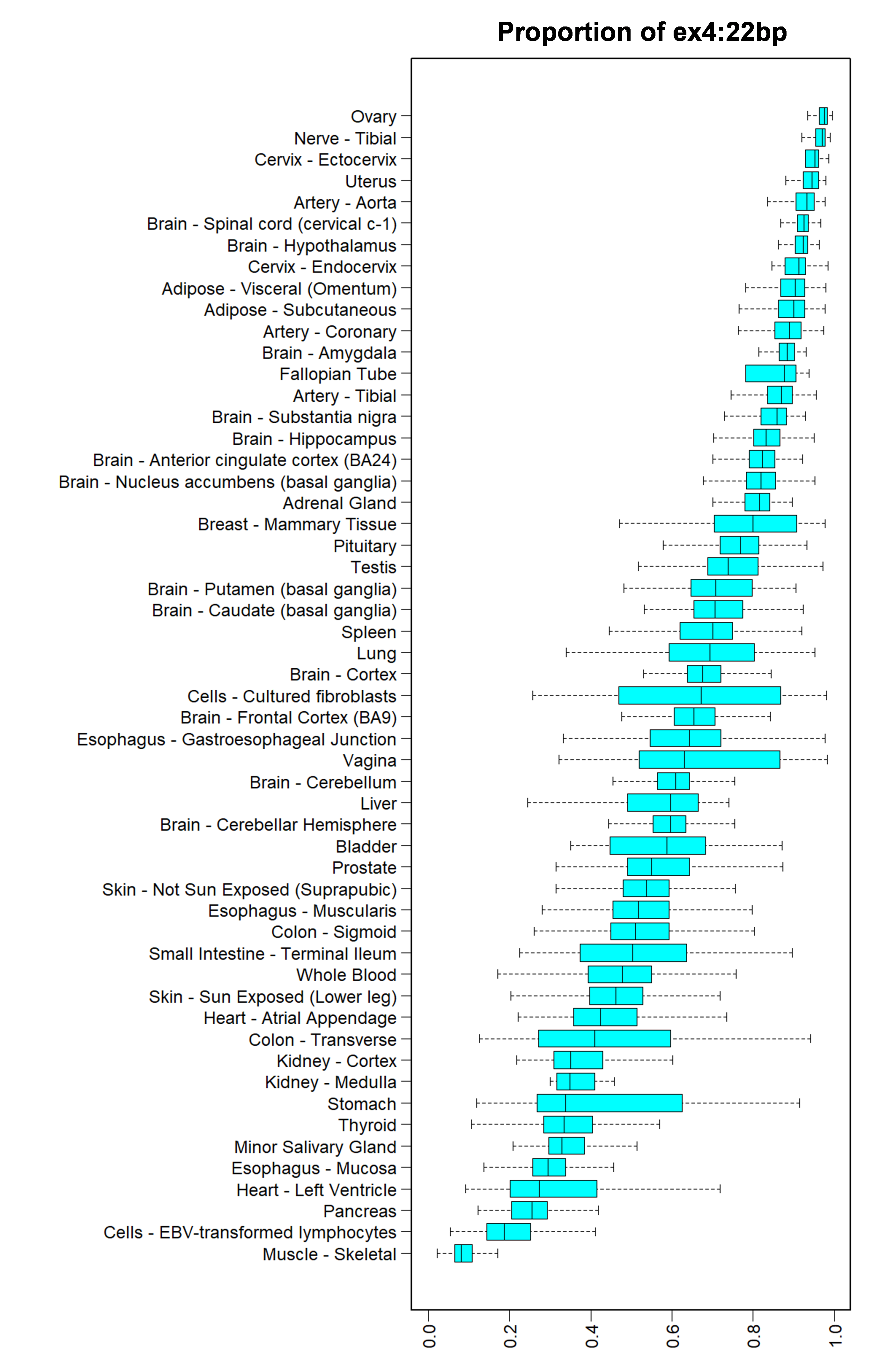

Supplement: Supplementary file 4 — Supplementary Figure 4. [file 41598_2024_63976_MOESM4_ESM.tif]

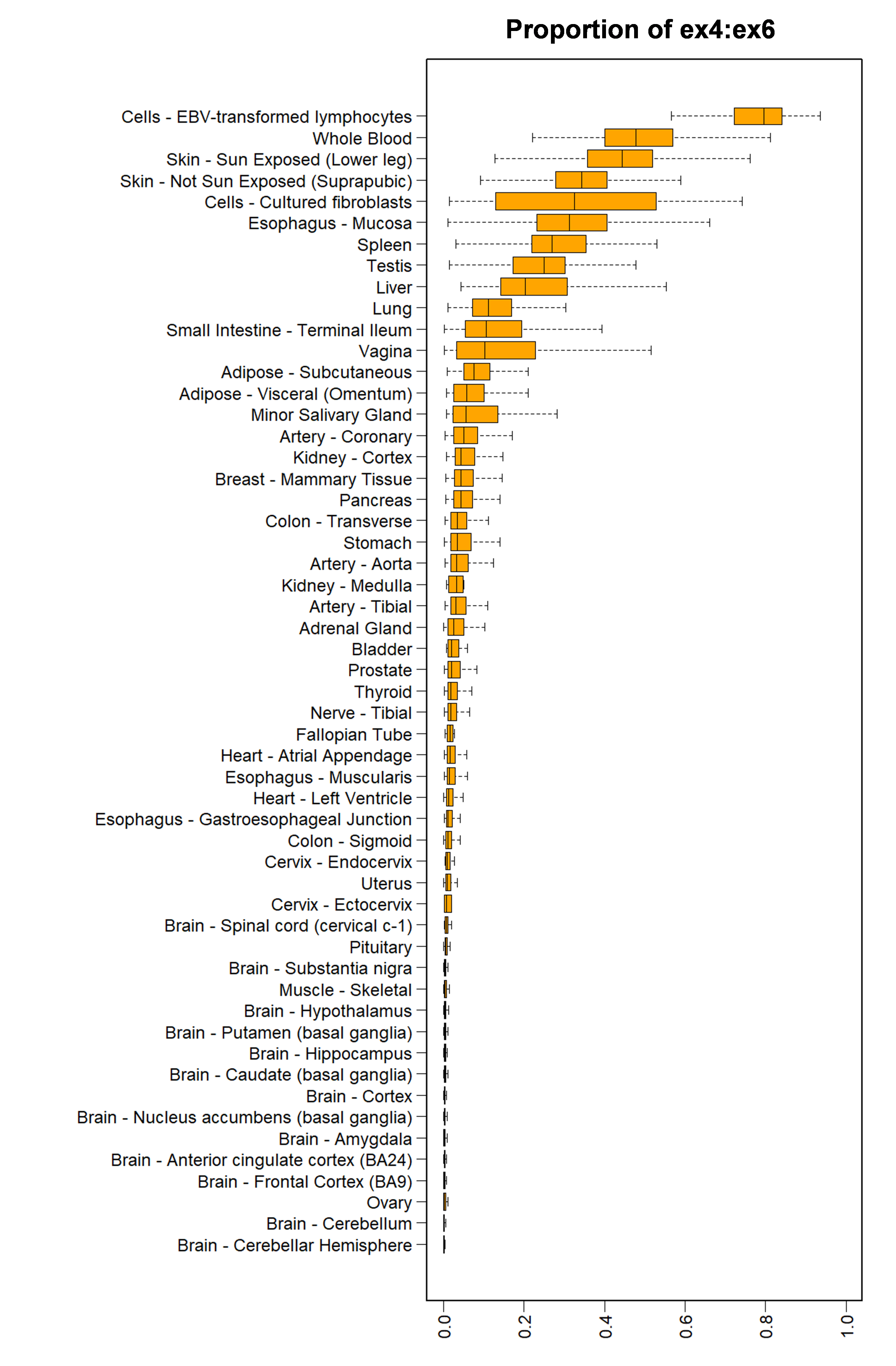

Supplement: Supplementary file 5 — Supplementary Figure 5. [file 41598_2024_63976_MOESM5_ESM.tif]

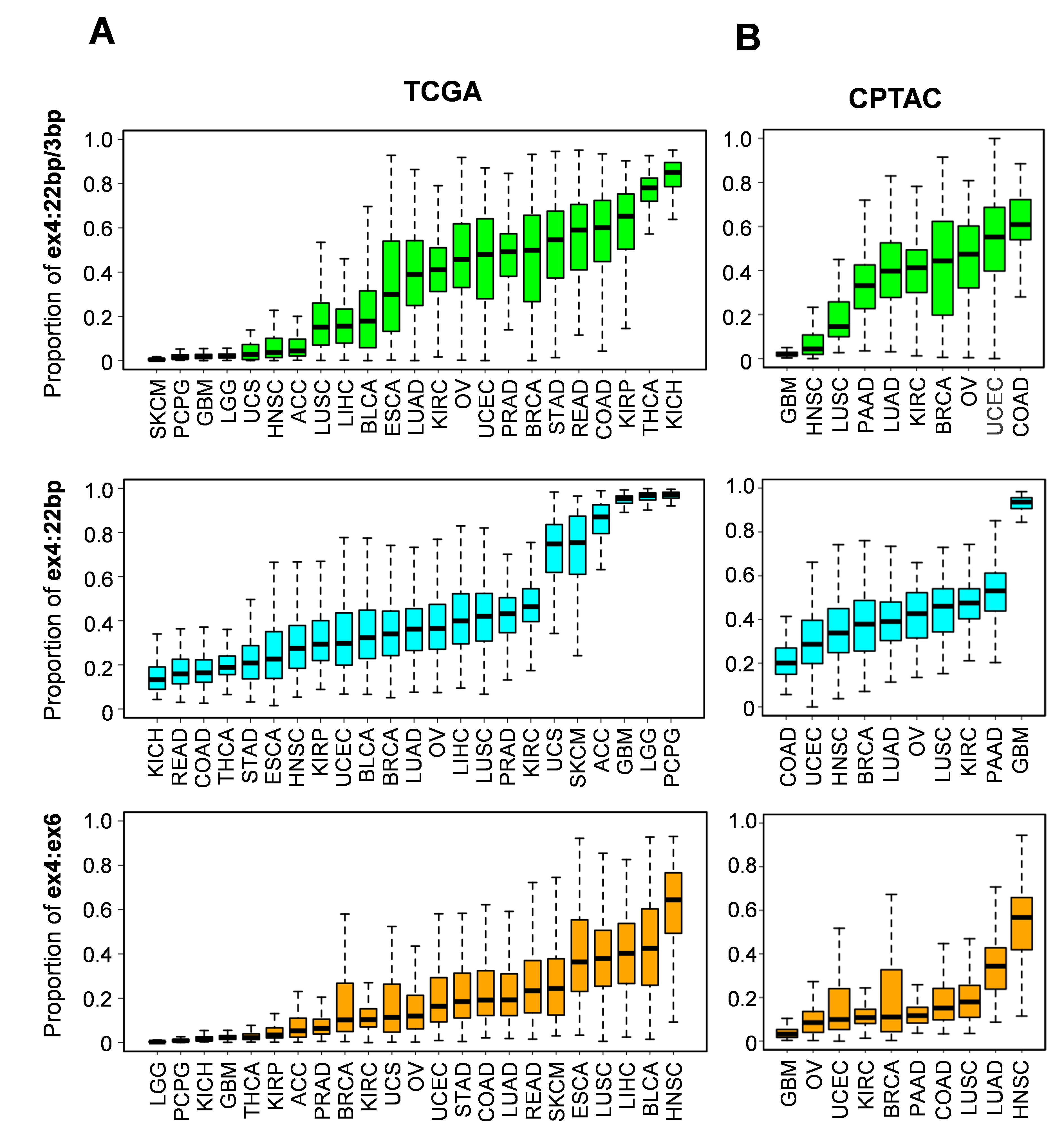

Supplement: Supplementary file 6 — Supplementary Figure 6. [file 41598_2024_63976_MOESM6_ESM.tif]

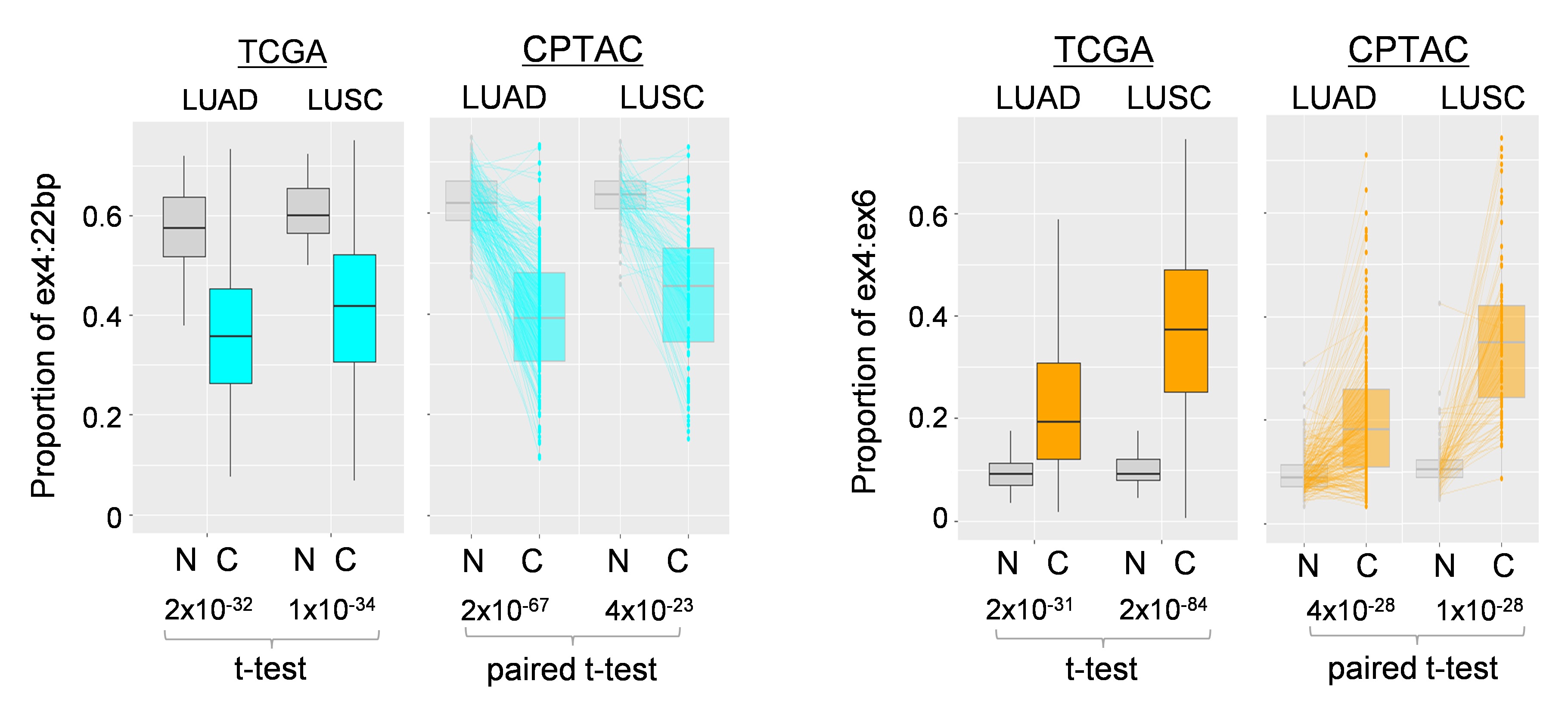

Supplement: Supplementary file 7 — Supplementary Figure 7. [file 41598_2024_63976_MOESM7_ESM.tif]
