# Supplementary material for: RPS24 alternative splicing is a marker of cancer progression and epithelial-mesenchymal transition
Source: Sci Rep. 2024 Jun 10;14:13246. doi: 10.1038/s41598-024-63976-y (PMC11162997; doi:10.1038/s41598-024-63976-y)
Supplement: Supplementary file 8 — Supplementary Legends. [file 41598_2024_63976_MOESM8_ESM.docx]

**SUPPLEMENTARY MATERIAL**

**Supplementary Figures**

**Supplementary Figure 1**. Prevalence of *RPS24* AS isoforms, calculated across total GTEx data. In total, 17,382 samples were used; 99.9% of junction reads originating from exon 4 belonged to these four isoforms.

**Supplementary Figures 2–5**. Diversity of proportion of *RPS24* AS isoforms across tissues in GTEx data. Supplementary Figures 2–5 show proportions of ex4:22bp/3bp, ex4:22bp/18bp, ex4:22bp, and ex4:ex6 in GTEx tissue, respectively. Tissues are arranged in order of median percentage of each isoform. Number of samples per tissue is shown in Supplementary Table 1.

**Supplementary Figure 6**. Relative abundance of each *RPS24* AS isoform in TCGA and CPTAC databases.

**Supplementary Figure 7**. Comparison of ex4:22bp (left) and ex4:ex6 (right) between cancer and normal samples from LUAD and LUSC in TCGA and CPTAC datasets. Since CPTAC dataset contains cancer and normal tissue pairs, we performed paired t-test for CPTAC data. Numbers of LUAD and LUSC samples are shown in Supplementary Tables 2 and 3.

**Supplementary Tables**

**Supplementary Table 1**. Names and number of samples in the GTEx dataset.

**Supplementary Table 2**. Abbreviations and full names of cancer types and number of samples in cancer and normal groups in TCGA.

**Supplementary Table 3**. General location and type of malignant disease and number of samples in cancer and normal groups in CPTAC.

**Supplementary Table 4**. Information from datasets obtained from Gene Expression Omnibus.

**Supplementary Tables 5–8**. GSEA categories were enriched among genes correlated with ex4:22bp/3bp, ex4:22bp/18bp, ex4:22bp, and ex4:ex6 junctions. Hallmark gene sets in MSigDB were used for analysis. Size: number of genes in each gene set. ES: enrichment score. NES: normalized enrichment score. NOM p-value: nominal p-value. FDR: false discovery rate.
